# From a Sociological Given Context to Changing Practice: Transforming Problematic Power Relations in Educational Organizations to Overcome Social Inequalities

**DOI:** 10.3389/fpsyg.2020.608502

**Published:** 2021-01-07

**Authors:** Yannick Lémonie, Vincent Grosstephan, Jean-Luc Tomás

**Affiliations:** ^1^CRTD, E.A. 4132, Conservatoire National des Arts et Métiers (CNAM), Paris, France; ^2^INSPÉ Reims, CEREP, Université de Reims Champagne-Ardenne, Reims, France

**Keywords:** Cultural-historical activity theory (CHAT), power relations, development, inequalities, interventionist research

## Abstract

In 2012, the international PISA survey reinforced the observation that the French educational system is one of the most unequal among OECD countries. The observation of serious inequalities in access to educational success for pupils from disadvantaged backgrounds could lead to a pessimistic vision suggesting that any possibility of transformation of the system is doomed to failure. Thus, the fight against inequalities in access to educational success is a form of runaway object which constitutes a challenge for research which treats the social context as evolving and susceptible to significant and novel transformations. Developmental work research aims to support the work of professionals in the re-elaboration of their practices by seeking to go beyond the status quo of an unequal school. Drawing on this framework within an institutional network of schools, we seek to show how the intervention has highlighted power issues inscribed in the structures and how the actors, through their commitment in the research collaborative process, seek to go beyond the power issues inscribed in their work routines and enacted during the research process by different kinds of antagonism. We will argue that the fight against educational inequality involves overcoming systemic power relations crystallized in institution. This systemic power is expressed by a form of episodic power. Our results show restrictive and constructive effect on the expansive learning process and on the construction of a collective in the formative interventions. The restrictive side of epistemic power should be linked to systemic power which is historically inherited. We discuss the results in the light of the emergence of a fourth generation of activity theory. Our research makes it possible to make conceptual and methodological progress in the construction of a fourth generation of activity theory by showing the need for analysis and expansively learn about problematic power relations in heterogeneous collectives.

## Introduction

Schools have an important responsibility in reproducing social inequalities. Numerous sociological works have shown the role played by structures and activities in the construction and reproduction of these social inequalities at school (e.g., Bourdieu and Passeron, 1970; Collins, 1971; Duru-Bellat, 2002; Teese and Lamb, 2007). To paraphrase Bronfenbrenner (1977, p. 528), it is one thing to understand and investigate the construction or reproduction of social inequalities at school. It is quite another to attempt to transform this reality and go beyond the *status quo*. Rooted in the tradition of the Cultural-Historical Activity Theory (CHAT), this article presents the first steps of interventionist research that accompanies the implementation of public policy aimed at reducing social inequalities in the access to educational success.

The anchoring of our research in CHAT departs from the conception of “sociological intervention” as it may have developed as a result of Touraine’s work (e.g., Touraine, 1984). Several differences exist between the intervention research carried out in CHAT and the sociological intervention method. Two main differences are worth mentioning here: (a) sociological intervention starts from a precise problem formalized by sociologists; whereas intervention in CHAT starts from a request made by professionals on the basis of concrete problems; (b) the objective of sociological intervention is to analyze the way in which individuals read and interpret the social world; whereas the objective of CHAT is to produce expansive learning to enable the development of activity systems. Finally, it should be pointed out that if, unlike CHAT, the theorizing of intervention has taken a back seat in Touraine’s work (Herreros, 2009; Cousin and Rui, 2011). Touraine’s methodological discourse suggests a sociology “in the middle of the ford” (Herreros, 2009, p. 57) between the production of academic knowledge and intervention. On the contrary, it is the transformations resulting from the intervention that make it possible to concretely understand the world and to produce knowledge within the framework of CHAT. For these reasons, our research departs from the tradition of sociological intervention.

More than 50 years ago, Bourdieu and Passeron (1970) highlighted the latent ideological functioning of the French educational system, which, in the name of its openly democratic recruitment, makes social selections based on the cultural criteria of the dominant class. For them, this social selection is made acceptable to the excluded and disadvantaged by a process of ideological production in which pupils from the working and middle classes find it necessary to legitimize their low social status through their failure at school. Half a century later, where do we stand?

In 2012, the international evaluation PISA^1^ points out that “*the correlation between socio-economic background and performance”* is much more pronounced in France, “*than in most other OECD countries*.” The French education system *“is more unequal in 2012 than it was 9 years earlier and social inequalities worsened mainly between 2003 and 2006. In France, when you belong to a disadvantaged environment, you clearly have less chance of success today than in 2003*” (OECD, 2012).

The school remains a powerful tool of hegemony in the sense of this concept as developed by Gramsci (1971). The concept of hegemony refers to “*a particular way of conceptualizing power which among other things emphasizes how power depends upon achieving consent or at least acquiescence rather than just having the resources to use force, and the importance of ideology in sustaining relations of power*” (Fairclough, 2003, p. 45). The observation of serious inequalities in access to educational success for pupils from disadvantaged social backgrounds could lead to a pessimistic vision, suggesting that any possibility of transformation of the system is doomed to fail (Engeström, 2017). However, for Sannino and Vaino (2015), who pointed out the importance of the concept of hegemony in a study on gender inequality in Finnish universities, “*dominance based on consensus is a contradictory phenomenon that is historically constructed and thus bound to transformations*” (p. 508). Accordingly, Kontinen (2013, p. 118) argued that “*the concept of hegemony is relevant to the contemporary developmental work research as well. First, it suggests paying attention not to economic relations but to political, institutional, ideological and cultural forms of power*.”

Two key points seem important to us here: on the one hand, it is a question of identifying the historical contradictions that make it possible to understand the construction and reproduction of educational inequalities; on the other hand, it is a question of re-inscribing the question of power in a developmental work of research in such a way as to make it possible to expansively overcome the historically constituted contradictions.

### Mode of Production of Social Inequalities in the French Educational System and the Struggle for Social Justice: A Brief History

The foundations of the republican school were laid in France at the end of the 19th century by the Ferry laws, affirming the right to education for everyone through compulsory schooling (Prost, 1968). However, in spite of the emancipatory project underlying the republican school, the school was divided according to social class: elementary school was the school of the people and secondary school, a school where the bourgeoisie formed the future elite (Dubet, 2019).

In 1918, a manifesto written by a group of officers taking the name of “Compagnons” was published. The Compagnons’ manifesto was the first step in a long historical journey to build a non-segregated school (Dubet, 2019). In the conception of the Compagnons, the idea of a non-segregated “single school” was intended to broaden the social recruitment of elites (Seguy, 2007). In this sense, it was a matter of replacing the social segregation of the two school orders (primary and secondary school) with a selection based on merit, regardless of the social milieu of origin.

Another conception of the single school took shape within the framework of the Langevin-Wallon plan in 1946 (Lelièvre, 2004). This ambitious reform plan, led by Paul Langevin and Henri Wallon, was never implemented but constituted an important step in the history of the French education system. It defended the idea of “*a total elevation of the nation regardless of the situation occupied in society*” (Lelièvre, 2004, p. 54). The idea of a single school was translated into the Haby law in 1975. This law led to an increase in the number of students in secondary schools, without impacting the original social environment for academic success. For Dubet (2019), the production of educational inequalities can be described as an interlocking of two logics: a structural effect, according to which educational inequalities reproduce socio-economic inequalities; and an effect linked to the organization and interactions at school that produce these inequalities.

In France, since 1959, schooling has been compulsory until the age of 16. Of French pupils, 83% attend schools in which schooling is free, where they are assigned according to their parents’ place of residence. Within this framework, some schools have higher proportions of pupils from disadvantaged neighborhoods than the national average.

The Priority Education (PE) policy was initially set up following the accession to power of a socialist government in 1981. The aim of this public policy was to correct the impact of social and economic inequalities on educational success by strengthening pedagogical and educational action in schools and establishments in areas with the greatest social difficulties.

Within different periods, several innovations have been attributed to this PE policy (Frandji and Rochex, 2011): (1) working with local partners in a network (local administrations of urban policies, cultural institutions, and social services, among others); (2) deconcentrating regulation and policy instruments as the policy of devolution of powers and administration is supposed to allow adaptation to local specificities; and (3) positive discrimination to give more to those who have less.

Two main contradictions can be found in this brief history: (1) between a project that emancipates the people through the school and an organization of the school into two orders that reproduce the social order; and (2) between an increase of pupil numbers in secondary education produced by the Haby Law and the selection of elites still inscribed in the ideological foundation of the French education system (Dubet, 2019).

### The Law of Rebuilding the School and the Reform of Priority Education

Following the results of PISA in 2012, the French government undertook, in the space of three years, a series of reforms aimed at improving the ranking of their schools in international rankings by implementing curriculum reforms, PE reforms, and a reform of school rhythms, among others. For the purposes of this paper, we will briefly present only two of these reforms: the reform of primary and secondary education cycles and the reform of PE.

**FIGURE 1:**
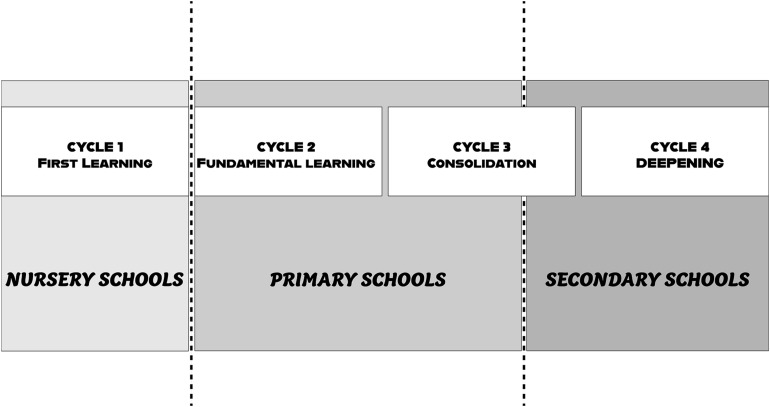
Organization of education cycles in French School since September 2016.

National educational curriculum has traditionally been organized by year. In the law “*on orientation and programming for the rebuilding of the school of the republic”* (July 2013), schooling was reorganized in a 3-year cycle of education. The curriculum referred to these cycles and aimed to allow greater flexibility in the work of curriculum development teams. The particularity of this reform was that Cycle 3 was shared between primary and secondary schools (Figure 1). In this context, the monitoring and establishment of a dialog between primary schools and secondary school seems important in the context of monitoring student performance.

**FIGURE 2:**
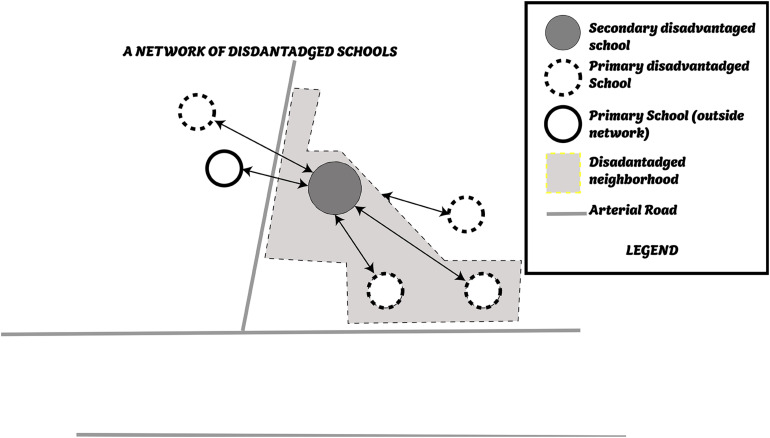
Geographical Representation of a Priority Education Network.

The second important reform concerns PE. The 2015 reform aimed to institutionalize the networking that emerged from the first PE policies. It was concretized by setting up the Priority Education Network (PEN). The PEN consisted of a secondary school and several primary and nursery schools that were part of its recruiting sector (Figure 2). Each PEN was “*managed”* jointly by an inspector in charge of primary schools and the director of the secondary school. The PEN’s animation policy was built and carried out within a steering committee whose geometry was variable. The actors of this steering committee, in addition to the two institutional pilots, were the network coordinator, network trainer, nursery, and sometimes, primary school directors.

The creation of these PENs aimed at the “*development of educational and teaching practices adapted to students’ needs’ and to enable evolution of educational organizations, specifically working better and more collectively as a team.*”

### Aims of the Article

The research on which the present research is based was part of the context of accompanying the refoundation of the 2015 PE policy. More specifically, the intervention research was initiated at the request of professionals confronted with networking and collective work between institutions not accustomed to working together. Two points must be emphasized concerning the contribution of this research.

First, while historic and sociological works made it possible to explain both the reproduction and production by education systems of educational and social inequalities, little work has sought to go beyond a comprehensive approach to engage in an interventionist approach that helps actors to learn how to construct their work organization to overcome the contradictions identified further upstream.

Second, the question of the reproduction or production of educational inequalities places at the heart of the analysis the question of the forms of power that is exercised in daily practices and likely to favor or hinder the expansive learning processes necessary for the development of work organizations.

In the following sections, we first describe the activity theory framework within which we operate and then explain how to conceptualize power in relation to the expansive learning processes involved in this interventionist research.

## Theoretical Framework

### Activity Theory as an Activist and Interventionist Theory

**FIGURE 3:**
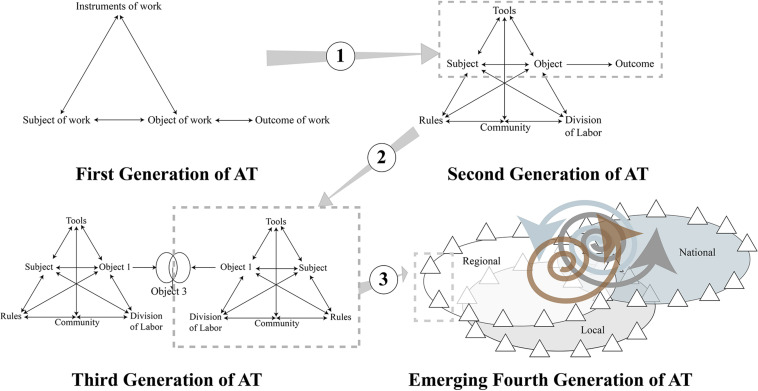
Four Generations of Activity Theory and Expansion of the Unit of Analysis.

Activity theory is generally presented in the form of three distinct generations (Engeström, 2001; Lompscher, 2006). Currently, a fourth generation is emerging (Engeström and Sannino, 2020; Sannino, 2020). The first generation of activity theory was built around Vygotsky’s notion of mediated individual action (Vygotsky, 1978), commonly represented as a triad composed of a subject, an object, and a mediating artifact (see Figure 3).

The focus on individual activity has been criticized. Thus, Engeström and Sannino (2020) asserted that one of the limits of this first generation “*is that it does not address social relations and the organizational anchoring of work actions*” (p. 4). According to them, this limitation was overcome by Leontiev’s definition of a system of activity organized into three hierarchical levels (the levels of collective activity, the levels of individual actions, and the level of operation) that are distinct and dialectically organized (Leontiev, 2009). Indeed, for Leontiev (Leontiev, 1976, p. 68), “*human work (.) is an originally social activity, based on the cooperation of individuals, which presupposes a technical division, even if embryonic, of the functions of work*.” Work as an object-oriented collective activity is carried out through actions directed toward a goal. In this framework, all individual action takes its meaning in collective activity. Thus, actions can be defined as processes whose goal does not coincide with the object of the collective activity. Starting from this example of primitive collective hunting, Engeström (1987, 2015) graphically represented a complex system of mediatized collective activities (Figure 1). In this second generation of activity theory, a collective activity system was taken as the main unit of analysis. Individual actions were considered as the “*tip of the iceberg”* (Engeström, 2001, p. 134), inscribed in a collective activity system that involves rules, community, and division of labor. To speak of an activity system refers to the idea that the elements of this system are interdependent, exist only in these interdependent relationships, and that the system constitutes the very context of mediated activity: “*In activity theory [.] contexts are systems of activity. The subsystem associated with the subject-mediator-object relationship exists as such only in relation to the other elements of the system. It is an in-depth relational vision of context”* (Cole, 1996, p. 141).

The globalization and internationalization of the second generation of CHAT pose the challenge of addressing issues of difference and dialog between multiple perspectives and the network of interacting activity systems (Engeström, 2001). Moreover, taking a collective activity system as the unit of analysis (in the second generation of AT) was clearly insufficient to explain societal phenomena, as “*activity systems do not exist in a vacuum, as they have been treated in second generation activity theory. They interact with networks of activity systems and are constitutive elements of them”* (Roth, 2001, p. 134). Therefore, the third generation of AT takes the interaction between multiple activity systems as the main unit of analysis:

*In activity theory, a collective activity system mediated by artifacts, seen in its network of other activity systems, is taken as the main unit of analysis. Individual and collective goal-oriented actions and groups of actions, as well as automatic operations, are relatively independent but subordinate units, ultimately understandable when interpreted in the context of the whole activity system* (Engeström, 2018, p. 14).

Various experts in activity theory have suggested the need to develop a fourth generation of theory (Lompscher, 2006; Engeström, 2009; Spinuzzi, 2020b). While some researchers advocate the need to consider new working conditions, especially in the era of digitization (Lompscher, 2006; Rückiem, 2009), the path that the founders of the third generation of AT (Engeström and Sannino, 2020; Sannino, 2020) chose to follow seems quite different. To align activity theory with current societal challenges (poverty alleviation and global warming, in particular), they broaden the notion of the object of activity through the concept of a “runaway object” borrowed from Giddens (2003), which is not only multi-voiced and fragmented, but contains global objects emerging from multiple and heterogeneous systems of activity whose positions *are ambiguous, and often seem to be subsumed by the object rather than controlling it*” (Engeström, 2009, p. 305). The challenge for a fourth generation of TA is therefore to achieve a true utopia (Wright, 2010) based on the articulation of formative intervention based on Change Laboratory (CL) methodology (Virkkunen and Newham, 2013) conducted with multiple and heterogeneous activities, actors, and institutions at different levels (local, regional, national, and possibly global) to address global problems. The present work is a modest contribution in line with this orientation.

Activity Theory (AT) is an activist and interventionist theory (Sannino, 2011). It aims to enable operators to be full actors in the transformation of work and its organization and, thus, in the creation of a new culture. For Engeström (2016), following the orientation of the historical-cultural theory of activity since Vygotsky (e.g., Stetsenko, 2016), human beings, whatever their age, are the creators of a new culture. This particular positioning of the CL makes it possible to question the dominant orthodoxy of constructivism, where individuals are seen as producers of their own development, rather than as co-producers of societal and cultural development (Roth, 2014). Ontologically, this position implies the consideration of the world as composed by collaborative practices that evolve historically and are constantly recreated in and by the actions of individuals (Roth, 2001; Stetsenko, 2015). At the epistemological level, it implies apprehending the knowledge process as part of collaborative practices that transform community practices.

The efforts of professionals in redesigning their systems of activity involve collective learning. This learning aims at transforming patterns of collective activity by transforming the very object of collective activity or the collective concept guiding collective activity to broaden the possibilities of action of professionals (Virkkunen and Ahonen, 2011). They are called expansive learning and are distinct from other forms of learning, traditionally viewed in terms of acquisition or participation metaphors (Sfard, 1998; Paavola et al., 2004). For Engeström and Sannino (2010, p. 2), these two metaphors of learning, despite their conceptual differences, share the same conservative presupposition and can say nothing about creating something new. Therefore, for Engeström (2016), expansive learning is the central mechanism for transforming societal practices and institutions.

In activity theory, the generating principle of expansive learning and the development of activity systems is recognition by professionals of contradictions within or between activity systems. These systemic contradictions must be differentiated from the dilemmas, problems, or conflicts that are merely symptoms (Engeström and Sannino, 2011; Ivaldi and Scaratti, 2018). The characteristics of a contradiction are that it can only be resolved by overcoming it or by inventing something new. Thus, formative interventions based on AT and expansive learning theory aim to enable operators to design their own activity system by giving meaning to the contradictions that bring the recurring problems experienced in their work.

In short, activity theory, and in particular the third generation, tries to overcome the dualism between agency and structure, between workplace learning and organizational and inter-organizational learning; it proposes to consider learning not as an individual process but to consider collectives and organizations as learners. At the same time, it proposes an intervention methodology to accelerate organizational learning processes by promoting expansive learning with the double stimulation method.

### Activity Theory and Power

Conducting formative interventions with actors from different institutions and at different hierarchical levels represents a risk if one does not consider, beyond the usual vertical division of labor, the different power relationships, latent and inscribed, in the culture and history of the institutions that are led to collaborate. The risk in ignoring these power relationships is that while qualitative change may occur within formative interventions, it is on the ground shaped by those who have the power to determine the changed agenda (Langemeyer and Roth, 2006). Formulated differently, if in the interventionist framework of activity theory, “[*p*]*ower becomes something that can be generated from below, by grasping the contradictions and by re-forging the activity to transcend the contradictions”* (Engeström and Sannino, 2020, p.8), it seems important to us not to ignore historically crystallized power relations in activity systems. It is a matter of questioning power relations that pose problems for gaining power collectively. Power is, in that sense a dialectical concept; it could be productive for expansive learning, as it could be restrictive by enable the protection of one’s own interest or hierarchical positions.

Yet, the issue of power and authority has very rarely been formalized in activity theory or organizational learning research (Blacker and McDonald, 2000; Blacker, 2009). Here we relay the question posed by Williams et al. (2018, p. 190): “[*w*]*hen we analyze an activity system, are we helping to resolve some of its contradictions and claiming to have made the system more effective, have we changed, transformed, or transgressed the power and structural relationships that matter?*”

Little research has taken up this question by mobilizing formative interventions as an instrument to work on power relations in the transformation of activity systems. Recently, Schirmer and Geithner (2018) analyzed, in an ethnographic work, the productive and restrictive effects of power to deal with contradictions. The productive and critical role of non-managerial actors is shown and highlighted by the authors. They also pointed out that future studies should examine how power models could themselves become the object of expansive learning. For us, this is a promising way of thinking about the development of organizations and activity systems in a less managerial, more democratic, and more open orientation in the actual conduct of the intervention.

Schirmer and Geithner (2018) highlighted that power is a multidimensional, contested, and relational concept and suggests four dimensions of power (Fleming and Spicer, 2014). They differentiate between episodic forms of power and systemic forms of power.

Systemic forms of power are, on the one hand, domination and, on the other hand, subjectification (Fleming and Spicer, 2014). For them, “*domination and subjectification are faces of power that can be considered systemic because they mobilize institutional, ideological, and discursive resources to influence organizational activity*” (p.240). These systemic forms are, if we relate them to the theory of activity, crystallized in institutions, tools, rules, divisions of labor, and constitute institutional, ideological, and discursive resources for organizational activity. Less visible than episodic power, this systemic power is, at the same time, historically constituted, and constitutes resources for the actions of professionals and can be apprehended through a fine ethnographic analysis of daily interactions. Domination is exercised through forms of ideological norms and is rarely questioned. Domination is a facet of power that shapes our ways of acting and thinking and our preferences, as well as our attitudes. It makes arbitrary hierarchical relationships appear inevitable, natural, and unquestionable. It provides a form of legitimacy to ways of doing things and confers legitimate authority on actors. It is rooted in a historically shaped organizational culture. In the framework of the French school, the hierarchy between a school of the people and a school of the bourgeoisie always weighs on forms of symbolic domination, which organize practices and necessarily impact the network of primary and secondary schools. Thus, the encyclopedic and elitist character of secondary school curricula, as well as the differences in status, salaries, and functions between primary and secondary school personnel, are rooted in the cultural history of the institution. Unquestioned, these forms of domination are likely to constitute obstacles to organizational learning (Bunderson and Reagans, 2011). Additionally, and within the French school, the selection of students on the basis of an evaluation and their orientation toward more or less prestigious courses of study constitute, from this point of view, a form of institutional domination that tends to legitimize the inequalities produced by the school. Thus, instead of social segregation based on two distinct structures as at the beginning of the 20^th^ century, a selection of students is substituted for a selection of students that makes the inequalities produced by the school more legitimate and less easy to question.

Subjectification constitutes a modern face of the exercise of power that is more creative and motivating than repressive (Nale and Lawlor, 2014). Rather than preventing the repression of something or someone, power is meant to incite and constitutes the subjectivity and identity of actors within institutions. In this sense, certain managerial innovations constitute technologies of power (Bardon and Josserand, 2018). Performance indicators, the need to improve the school, to anchor teaching methods on scientific and experimental bases (Evidence-Based Education), constitute dominant discourses, which, once appropriated, constitute the very identity of the actors by allowing them to appear in a favorable light in the hierarchical chain.

From the perspective of episodic power, coercion is the direct exercise of power. This direct exercise of power is exercised through the action of coercing someone to do something expected of them. Fleming and Spicer (2014) noted that individuals tend to go beyond the legitimate authority given to them. Coercion is more particularly visible in conflicts; the settlement of which requires submission to the majority or to hierarchical authority (Engeström and Sannino, 2011). Less visible manipulation is exercised through an implicit shaping of issues considered implicit or relevant. For Fleming and Spicer (2007, p. 17), “*there is no direct exercise of coercion here. On the contrary, there is an implicit shaping of the issues considered important or relevant.*” Thus, manipulation is exercised, for example, in the construction of the agenda of a working meeting where only those questions that appear legitimate are dealt with.

Episodic forms of power exercised in everyday work situations represent both symptoms of underlying contradictions within activity systems and a potential lever for their transformation (Engeström and Sannino, 2011). In this framework, our hypothesis is that the identification of these forms of exercising power constitutes, within formative interventions, a possible path for actors to discuss and question the systemic forms of power crystallized in institutions.

**FIGURE 4:**
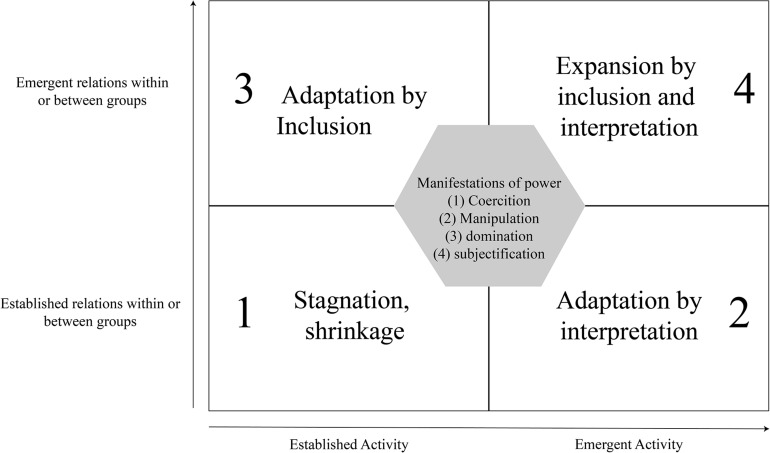
A framework to analyze power in expansive learning processes from Schirmer and Geithner (2018) and Blacker and McDonald (2000).

Within these formative interventions (Schirmer and Geithner, 2018, p. 20) note that “*power relations both allow and hinder the journey across the terrain of the zone proximal development leading to more (or less) expansion in terms of divergent interpretations and social inclusion of divergent actors*.” Blacker and McDonald (2000) attempted to model organizational learning in the form of a quadrant diagram constructed from two axes (Figure 4). The horizontal axis distinguishes between established and emergent activities, and the vertical axis distinguishes between established relationships within or between groups and emerging relationships within or between groups. For the authors, organizational learning is movement between these quadrants. Schirmer and Geithner (2018) exemplified this movement in a case study within a molecular biology company. The authors used a comprehensive approach that analyzed this movement. They noted (p. 31) that “*future studies should particularly show how the patterns of power and mastery could themselves become the object of learning*.” The intervention of which we present some aspects is in line with this orientation.

## Materials and Methods

### Collecting Mirror Data and the First Step in Formative Intervention

The request was initiated by the Créteil and Reims school regions. Fourteen NDS were involved in this formative intervention. The objective of this formative approach was to help professionals in the implementation and management of priority education networks. In both regions, the initiative for the approach was taken by the managers of these 14 networks, which were then relayed by the rectorates of each of the two administrative regions.

The implemented approach involved a research-intervention consortium, mobilizing several laboratories. The work was carried out by alternating local work within each PEN and grouping sequence by administrative region (Reims and Créteil, respectively).

The protocol was largely inspired by the CL methodology (Virkkunen and Newham, 2013), while combining it with contributions from the “*clinics of activity”* methods of self-confrontation interviews (Clot and Kostulski, 2011; Kloetzer et al., 2015). The work was carried out by alternating the collection of mirror data on different sites, collective work times within the networks, and network grouping times, where inter-network and intra-industry work could be carried out. In formative intervention, mirror data are collected for team reflections and analyses. These data can be videos, documents, figures, map, statistics about problematic situations, disturbances, and innovative solutions. These mirror data aim to analyze the contradictions behind problematic situations experienced during everyday work in formative intervention.

Our data collection consisted first of an audio-video recording of a steering meeting in each network and then of a self-confrontation of the different actors present in the recorded meeting. The objective of the interviewer was to access everyone’s “*real of activity*,” beyond the “*realized activity*.” The real of activity includes what we wanted to do, what we couldn’t do, what we do for a while, what continues to inhabit the present situations, what we do to avoid doing what is to be done, and what we don’t do (Clot, 2006). In other words, it was a question of questioning the different actors to understand the conflicts at the heart of the action of these professionals.

We supplemented this data with audio-video recording data of sequences of debates and discussions within the steering committee. These discussions were organized based on tools produced by the participants, such as a diagram or a network map. The main objective of that work was also to access the perception of activity and could be seen as a crossed self-confrontation interview (Clot et al., 2002).

Finally, we inferred a first hypothesis of the main contradictions from the analysis of the problems, tensions, and dilemmas that appeared during the steering committee. This analysis was based on particular self-confrontation data.

### Double Stimulation as an Expansive Learning Generator in Formative Interventions: Second Step of Formative Intervention

The second stage of the formative intervention mobilized data from the first phase as mirror data. We selected data by anonymizing it in such a way to allow each of the networks to focus on problems that were relatively similar to those that other networks had encountered. Two criteria were important in this context: (1) that each of the networks could identify themselves in the mirror data and (2) that the work could be done without personifying the problems encountered, which we felt would have been the case if we had not adopted this procedure.

In this second phase of the formative intervention, the work was more closely linked with the methodology of CL. Each of the workshops was recorded for later analysis. The work was still in progress at this level as it had to be interrupted for health reasons and in view of the additional workload linked to the reopening of schools.

The research data used in this article consisted of excerpts from audio-video recordings of two sessions of a workshop conducted with a collective of professionals from a priority education network in the city of Reims, France. This collective was made up of the network’s three institutional managers (the director of the secondary school, the primary school inspector of the district to which the network’s nursery and primary schools belong, and the secondary school inspector), the network coordinator, priority education trainers, and the directors of the network’s nursery and primary schools. Therefore, it was a heterogeneous collective, made up of members from different levels of education, occupations, and organizations. The workshops were conducted in an orientation close to the Boundary-Crossing Change Laboratory.

In this workshop, the data collected were intended to reflect a developmental process brought about by the so-called double stimulation method (Sannino, 2015a,b). This method aimed to support the process of social remediation (Virkkunen and Schaupp, 2008) implemented as part of the developmental intervention as we deployed it. Initially conceived as a particular method of experimental investigation using two sets of stimuli, the double stimulation process referred to the mechanism by which human beings can deliberately emerge from a conflict situation and change their circumstances or solve problems. The first stimulus had the function of a task toward which the activity of the experimental subject was directed, while the other takes on the function of signs that help to organize the activity. In the context of a developmental intervention, the first stimulus consisted of placing the actors in front of a problematic situation in their work that provokes a “*conflict of motives”* and leads the subjects to analyze, characterize, and interpret the problem. This conflict “*is resolved by using a neutral artifact as a second or auxiliary stimulus which, through the meaning it is invested with, is transformed into a mediating sign*” (p.6), which then allows us to think and conceptualize the transformation to come.

The sequential analysis of the exchanges made it possible to uncover the dynamics of the construction of the problem and its relevance in becoming an object of work in the framework of the developmental intervention. It aimed to identify the discursive manifestations of power relations between the different protagonists and their movements.

## Results

### Data Expressing Power Relations That Matter

#### Conflict and Coercion During a Steering Committee

The conflict that emerged within the steering committee meeting concerned the setting up of pedagogical action by the network coordinator involving primary school teachers and secondary school mathematics teachers (Table 1). The director of the secondary school opposed the participation of secondary school teachers because, although informed, the director must plan and organize the work of teachers. The inspector of the first level, who is the hierarchical superior of the coordinator, sought to be the mediator of this conflict and pointed out several times that the rules were different between the first and second levels; if it is possible to deal directly with teachers in the first level, it is not possible to do so with the second level, and it is necessary to discuss it in advance with the management.

**TABLE 1:** Excerpt from a Conflict During a Steering Committee Meeting of a Priority Education Network.

**Coordinator:** We received an email from the mathematics teacher. So, five teachers want to participate in the math challenge that concerns all schools of the town. And so, the next steering committee will be on December 4, all day and so I will send at the same time as the teachers, to the teachers of your school (address to the director). (.) They seemed very interested, very involved, so they will be invited to this working time on December 4. So yes, they are invited. They are given time slots and they come whenever they want. **Secondary School Director:** Oh yes.but no! You have to check with us before. It’s not that we’re against this kind of meeting, but we need to know as soon as possible to know when they won’t be there in the establishment. **Primary Schools Inspector:** I think the idea there was about their off-class hours. But it is indeed necessary that the direction is in the loop so that you can’t it organize yourself. **Secondary School Director:** (.) So that you understand, teachers are not free electrons. **Coordinator:** So it is up to you to define their availability that day? **Secondary School Director:** That’s not what I’m saying either. You talked about the fact that you’re going to offer them several slots… **Coordinator:** But it’s all day long. **Primary Schools Inspector:** But in fact, you have to talk with the secondary school director before. **Secondary School Director:** Yes! **Primary Schools Inspector:** It is. it is absolutely essential that you (the director) are in the loop, that you be solicited for these questions. Because so that this does not disrupt the secondary school and at the same time it is a joint institutional work, you (the coordinator) really need to be aware of it. **Coordinator:** But it seems to me that you were in the loop of the email that the math teacher coordinator sent. **Secondary School Director:** I received that email, but then. **Primary Schools Inspector:** In fact, you should not deal directly with the teacher. In the first degree at least, you can do it without going directly through the directors. But for the secondary school, you have to talk with the director before. **Coordinator:** But. **Secondary School Director:** Listen, send me the email and then let me handle it **Coordinator:** Okay. **Secondary School Director:** Because our goal will be to make this connection possible.


All the actors involved, without exception, spoke about this episode in their self-confrontation interviews following the steering committee. The coordinator and the director of the secondary school did not share the same object of work on the network. For the director, it was a question of working on “*the articulation between the levels of education*.” More specifically, it was a question of working on the articulation between primary and secondary schools, which explains why the agenda of the steering committee concerned cycle 3 almost exclusively. In contrast, for the coordinator, it is a question of “*working on new pedagogical modalities with primary and secondary school teachers who are not used to working together*.” While the coordinator was trying to create a different space for teachers to collaborate, the secondary school director was trying to control the actions implemented on the network insofar as they must mainly concern cycle 3.

This conflict illustrated the resistance inherent in the interaction between different activity systems to the networking of disadvantaged institutions. This conflict was resolved by coercive action on the part of the high school principal. The director of the secondary school and primary school’s inspector, who are institutionally the managers of the PEN, sought to maintain the authority of which they were institutionally responsible. The most interesting thing in this excerpt was the reminder of the rules that are not the same in primary and secondary school. While the coordinator must inform the secondary school principal in advance, he does not need to inform the directors of the elementary school in advance. The rules change according to the level of the institution and reflect a form of hierarchy between primary and secondary schools. This form of hierarchy, which reproduces the two orders of teaching identified in the history of the school institution, is both a resource for the action of the secondary school principal to control the work at his school, as well as a hindrance to the work of the coordinator.

This form of hierarchy was reinforced by the fact that the headteacher exercises a form of coercion that goes far beyond the legitimate authority conferred on him. In doing so, the network coordinator finds it impossible to carry out the work; it is a question of organizing schoolwork in new forms in a dedicated collaborative space where teachers work in horizontal collaboration. The director of the secondary school had a direct hierarchical authority over teachers, an authority he did not intend to grant: “*At our level, we are the ones who make the changes in secondary school. Mastery of change (.) and work materials are essential to be able to control this change. At the network level, we direct the change, that is to say the inspector and me, but after it is not, we, who will implement the actions that we suggest*.” The change in control seems very important for the managers and they attempted to maintain their authority to enable this control in the network. This explains why the director exercised a coactive form of power over the coordinator, although they were not hierarchically in that vertical division of labor. The coordinator had to develop the collaborative work in the network without having authority: “*We, coordinators, are the converging centre of conflicting interest.*”

As shown in Figure 4, this form of coercion, beyond impeding the coordinator’s work, constituted a hindrance to collective work on the network, insofar as the coordinator was attempting to create a third party space, independent of the distinctions between primary and secondary schools to make teachers and students work differently. For the coordinator, the object of the work consists “*to offer teachers of different school levels different working arrangements that engage them together. And projects are always a different modality. So suddenly things make sense for the students*.”

#### Manipulation: Harmonization Versus Achieving One’s Own Goal

In a different network steering committee meeting, the vice-director of the secondary schools asked for the harmonization of the student skills assessment sheet that enables transmission of information between primary and secondary schools. During the self-confrontation interview, the vice-director said that it was necessary “*to harmonize form between schools to be able to constitute homogeneous classes at the beginning of the year.*” This was not presented as a justification of that request during the steering committee. The request was justified by the fact that the forms were filled in by teachers in a very heterogeneous way; while some teachers evaluated by grade, others referred to the skills acquired by students.

For the director of the primary schools, this request was badly felt. During the following self-confrontation interviews, they expressed that: « *as usual it is a demand from the secondary school* »; « *we have to do the job three times: first for the institution, second for the student’s parents, third for the college* ». They explained that it is difficult for them to force their colleagues to harmonize their assessment practices because, in the French system, the director of a primary school is a function and not a job; they do not have the authority to order their colleagues to change their assessment practices. They also expressed a kind of domination from the secondary school direction; they must work together, but they do not have the opportunity to express their own concerns and needs. As in the first example, the focus of the direction of the secondary school concerns cycle 3 and the articulation between primary and secondary schools. This focus on the articulation between primary and secondary education was again an obstacle to the development of collective work.

The inspector of primary schools understood that « *all teachers do not respect the institutional rules of evaluation: some continue to evaluate with grades whereas they should evaluate skills ».*

In this way, directors of nursery schools try to find their place in a conversation from which they feel excluded: « *It’s always like this: no demand comes to us. While we have competencies to participate to such task ».*

The fact that the object for the direction of the secondary school is to work between the level of teaching implies that all the meeting is to focus on the cycle 3 (Figure 1). This articulation is also problematic because of institutional differences between primary and secondary schools.

One of the participants explained that “*personally, I think that the reform of the college wants to make the famous fundamental school of the post-war Langevin-Wallon plan. There is an insurmountable problem in national education, which is the status of primary and secondary school teachers. In terms of contradiction, they are trying to steer us toward a 6-year-old school of 16 years old with completely different status, the culture of the second degree will never be able to function like the first degree. We pilot with opposing energies that are institutional.*” The different history of primary and secondary school explains differences in the status, education, and training of teachers. These different histories explain a kind of symbolic domination from the secondary school under primary schools expressed in a vertical management of the direction of the secondary school and in the choice to work collectively on tasks closed to the interest of the secondary school.

#### From Symptoms to Contradictions: Implementing a Network in a Hierarchical System

**FIGURE 5:**
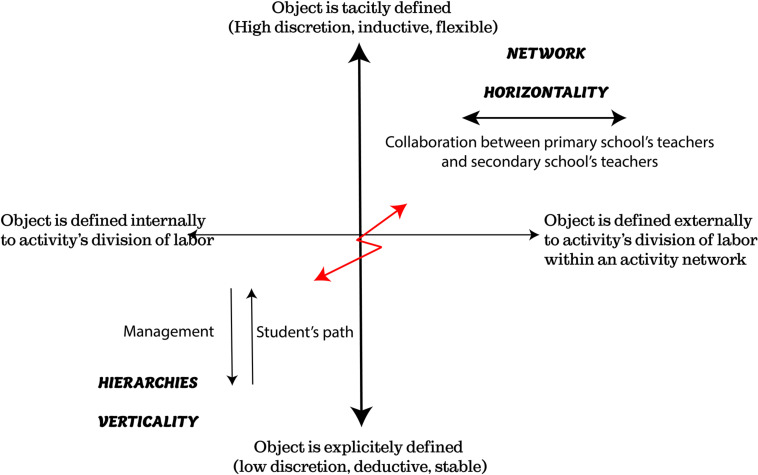
Contradiction Created by the construction of a PEN in a hierarchical system, adapted from Spinuzzi (2015).

Figure 5, based on the work of Spinuzzi (2015), represents the contradiction between the implementation of the reform embodied by the activity of the coordinator (whose function was created in the reform) and the activity of the principals who seek to maintain control. The reform that promotes network collaboration comes up against a system that is hierarchically and vertically organized by the student’s path and by the way in which it is managed. If power is expressed through coercion and manipulation, it is rooted in a form of domination of the secondary over the primary.

Multiple other cases illustrated within our work in networks, this form of symbolic and hierarchical domination between the two orders of teaching. They result in difficulties to work in a collaborative way and to build a horizontal workspace that transcends the verticality of the students’ path within the school institution.

### Second Step of the Intervention: Constructing the First Stimulus

The first stimulus here refers to the recognition of problems requiring a transformation of the steering activity. However, this recognition is not self-evident and is not spontaneous. It presupposes a triggering element for the possibility of such a recognition process to occur. This triggering element is a time when all the voluntary networks of the Reims Academy are brought together during which an intra-trade work was organized (director of secondary school, directors of primary schools, primary schools inspector, network coordinator, vocational trainers). The requested work consisted of reacting to extracts from verbatim extracts of interviews conducted with people working in the same profession as them in other academies. The aim was to express agreement or disagreement with these statements and to launch a debate within the group based on these agreements/disagreements. The group then produced a synthesis of the key points discussed and presented it to the other groups. This work was a trigger for the recognition of a problem in that the primary school inspector of one network wanted to use the presentation of the primary school director’s group to address a power issue in a workshop session within this network. The following excerpt illustrates how the issue was introduced by the primary school inspector at this session.

**Primary School Inspector:** What was interesting on the afternoon of 7 March, when we had the inter-trade working groups, Mr. X, you took part in them and then you too A. I don’t know what you thought about it, I found it interesting when we had this work on concerns. We had an inter-trade working group, we had the concerns of the managers and then the concerns of the coordinators within the network, what were their concerns as directors of secondary school, and then there was this synthesis. And I found this very interesting precisely because we realized, on the return of the school headmasters, their view of what they could express about the pilot and how they perceived it, and I found this very, very formative for the occasion.

**Director of the Secondary School:** What came out of it? I wasn’t there.

**Primary School Inspector:** Precisely this idea that, although we may think we share a common culture and we try not to be too vertical, descending, in fact there is always this hierarchical distance that makes it there,

precisely the word of one is not equal to the word of the other and I found that very interesting. Is the word of a pilot worth the word of a school headmaster or a coordinator? The feeling was actually there. No, in fact, as a coordinator, as a school headmaster you don’t have that feeling, my word is not worth the word of a pilot, whereas if we were on a pilot, I don’t know what we could call it, shared, horizontal. In the best world, the word of one would be worth the word of the other.

However, the introduction of this subject of discussion by one of the 3 pilots of the network, did not yet mean a shared recognition of a problem, as the first reactions of another pilot showed.

**Director of the Secondary School:** It’s a bit in that spirit that we are when we have a steering committee meeting,

**Primary School Inspector:** I don’t think so.

**Director of the Secondary School:** That’s how it feels, that’s how I feel. Everyone is a force for proposals.

**Primary School Inspector:** In the end, we always make the decisions, and we had prepared the meeting.

**Director of the Secondary School:** Yeah.

**Primary School Inspector:** We come, we have proposals and then.

**Director of the Secondary School:** We may be at the source of how it will happen.

Then, the directors of primary schools’ reactions were timid and tended to be more in the direction of astonishment as expressed by the headteacher. Teachers did not allow themselves to be more involved in policy making, even doubting its legitimacy, as can be seen in the following excerpt:

**Director of Primary School 2:** Sometimes we expect it too, at last. I don’t know if I can illustrate this with the example of the Cycle 3 council the other day [.] we don’t know if we intervene, we ask for your approval, we make a proposal regarding the inter-degree course, regarding Delphine’s answers, and there I don’t feel comfortable [.] I don’t feel legitimate.

I don’t feel legitimate. I don’t feel legitimate. I don’t feel legitimate. I don’t feel legitimate.

**Director of Primary School 3:** Me neither.

**Primary School Inspector:** It’s coming, it’s coming, there is the emergence of the fact that the word, the point of view, the word of the field, of a director of the primary schools are taken into account in the training. I think that this becomes interesting, because it contributes to the appropriation of a common culture, that is to say that at the beginning it was not necessarily the direction that would have been given.

In the rest of the discussion, this issue was put to work at this meeting without these “habits” being fundamentally challenged by the headmasters themselves as the inspector of the primary schools tried to shake them up.

**Researcher:** In the workshop with the headmasters it was a real question as to whether or not they feel they are pilots or not?

**Primary School Inspector:** Do they in fact allow themselves? Do they allow themselves, that’s it?

**Researcher:** It’s very important what you’re saying here about the question of authorization, for example, do we allow ourselves to take initiatives?

**Primary School Inspector:** Do we authorize them? Yes, not everyone authorizes themselves. **Coordinator:** We are used to living in a very hierarchical society, we must live with.

However, the insistence of the primary school inspector allowed the school headmaster to explicitly express a problem that they did not dare to raise until now.

[h] **Director of Primary School 2:** [.] for the steering, what is complicated is that we are often the link in something that goes down and there that is really very vertical and where we have to make it become horizontal, to try to get the team’s support. It’s true that it’s not easy, yes, it’s not easy.

**District Pedagogical Adviser:** that’s it, and then the status of primary school directors is not a status.

**Director of Primary School 1:** And then we are on the last rung too, that is to say that we also have the same concerns as each teacher [.] And then we are alone in the end too. In a school we are the only ones, and at the same time we have the double role since we have our class and we also have this role (of director). It’s true that it’s not the same in secondary school where there are several actors, administrative, pedagogical. It’s not necessarily always the same person who will propose something. But it’s true that it comes from the secondary school, from the primary schools inspectors, from the educational advisers, in the end it’s always the same person who will centralize the matter and who will have to make the whole thing public.

Thus, these statements express the fact that headmasters are the only place where the different prescriptions they are responsible for are transmitted to the teachers in their school converge. On the one hand, they have not been involved in defining them and, on the other, unlike the principal of a college, they do not have hierarchical status vis-à-vis their colleagues.

The work carried out with the researchers initially led to the inclusion of school headmasters in the reflection on piloting, which was not initially envisaged by the pilots. This had the effect of questioning the pilots about the perception of school headmasters in the steering methods used in this network. This awareness, examples of which we have just seen in the previous paragraphs, also led to a positive evaluation by the school headmasters of this work of reflection, as shown in the following extract from the same meeting:

**Director of Primary School 1** : [.] It’s true that all these meetings make me, personally, feel more in charge because I understand more things, because we hear more things too. Afterwards, there are still times when we are not present and therefore there are still areas of uncertainty.

[…]

**Director of the Secondary School:** Perhaps in the future we can also envisage a slightly more logical consultation, i.e., upstream rather than downstream, because it’s true that when we set up our steering committees, we are in a very small committee, we’re not going to start laying down milestones among ourselves and imagining avenues of work. Perhaps we should.

**Primary Schools Inspector:** that it should be more participatory in fact.

**Director of the Secondary School:** There you go.

The work described here is in progress and we have no information at the time of writing about how the process will continue. In any case, the intentions were stated and shared by all the actors of this network involved in the IR project.

The different sequences presented above showed that issues of power and hierarchy are not immediately perceived as problems or obstacles to effective networking, but as a “*normal*,” almost “*natural*” situation. This perception is a symptom of cultural hegemony that has succeeded in imposing a form of steering based on an immutable vertical division of labor, which contradicts the participatory aims associated with networking. The intervention, by means of the first stimulus, made it possible to discuss this conflict of motives: hegemony vs. natural situation. It also presented the possibility of questioning a habitual process of exercising steering and reflection for the development of new forms and organization of work.

## Discussion-Conclusion

### The Dual Function of Power in Expansive Learning

Our results, in line with Schirmer and Geithner (2018), showed that episodic power has a dual function in expansive learning. In the first stage of the formative intervention, the results showed that forms of coercion and manipulation constituted obstacles to the collective work. More specifically, the PE policy was translated into the creation of a collective workspace (the meetings of the steering committee of each PEN). However, within these networks, the creation of a true collective work is prevented by the exercise of an episodic form of power. In the first case, the form of collective work proposed by the coordinator was compromised by the coercion of a director of the secondary school. This form of exercise of power was justified for the director of the secondary school by the authority he has over his own teachers. As he himself puts it, “*Teachers are not free electrons.*” However, this form of coercion, which partly resolved the conflict, exceeded the legitimacy of his position. Indeed, the coordinator was not within the same hierarchical line. In this episode, coercion was used to safeguard one’s own interests and one’s own territory, which was that of the secondary school.

Defense of the secondary school’s interests was also translated in forms of manipulation, which was justified by an impoverished form of the object of the network, as the management of the secondary schools thought it was; for them, the work on the network was a work on the articulation between the schools within cycle 3. For them, work on the network was adjusting the articulation between schools within cycle 3. This focus on cycle 3 in all the selection committees reflected a form of symbolic domination that found its distant origins in the history of the French education system. The elementary school was the school of the people and secondary school was the school of the bourgeoisie. More particularly, the statutes were different and if the secondary school was endowed with a direction, within the framework of the elementary school, it was a function occupied by a voluntary teacher, discharged from a part of that teaching. The status of teachers also differed; primary teachers were generalists, whereas secondary school teachers were subject specialists of a discipline. Therefore, manipulation consisted of presenting issues that appear legitimate for secondary school without really recognizing the specific needs of primary or nursery schools. The effects of these forms of manipulation were particularly badly experienced by directors of nursery schools, who were invited to participate in a discussion that does not really concern them.

In the two cases, episodic power had a systemic form of power crystallized in the institution. The modeling provided by Schirmer and Geithner (2018) made it possible to consider the “episodic” power of actors, in its political and relational dimension, in the sense that it depended on actions between subjects, and the “systemic” power of organizations, which did not depend on direct actions between actors and was part of a more general phenomena of structural domination and subjectivation, such as the process of internalization of hierarchy as a “natural” situation (cf. the Gramscian approach to hegemony mentioned above). Nevertheless, the third example seemed to show a potentially positive effect of the exercise on an episodic form of power. The initiative taken by the primary inspector to bring this vertical division of steering work into discussion with the other players in the steering process, particularly school headmasters, made it possible to envisage new forms for the processes of instruction and decision-making on the network’s files. By resituating their modeling within the framework of an expansive learning process, Schirmer and Geithner (2018), inspired by Blacker and McDonald (2000), proposed a framework for analyzing power relations and their potential development (Figure 4), which is heuristic for analyzing this example. This framework was organized around two dimensions: a dimension relating to the emergence of relations within or between groups and a dimension relating to the emergence of collective activity, its objects, and the models of interpretation (meaning) mobilized. Expansive learning can then be a non-linear movement between the four quadrants of the model.

Based on this schematization, we revealed non-linear movement between the quadrants of the model. The first process of inclusion (from quadrant 1 to quadrant 3) was identified. It was characterized by the expansion of the focus group to include primary school directors, who were previously excluded. However, this process of broadening the scope of the actors, the social relations between these actors, and the multiplicity of voices was only made possible by the willingness of the pilots. It was, therefore, an episodic exercise of power that had positive effects. However, expansive learning also implies allowing the expression of a diversity of conceptions, meanings, and ways of seeing (process of interpretation, from quadrant 3 to quadrant 4), which was also made possible by the action of the primary school inspector, who after having wanted the presence of the primary school directors, wished to give them a voice but also a value that had not previously been fulfilled.

These two movements (inclusion and interpretation) were made possible by an episodic exercise of power, whereas other examples in other networks, where the presence of actors other than the pilots was not desired, seemed to show that the dominance of a systemic form of power (domination/exclusion and subjectification) would rather have restrictive effects on the development of steering activity, leading to stagnation or even a shrinking of this activity.

### Generalization as a Process Transcending the Level of Formative Intervention

Here, we discuss the question of generalization of intervention and the results that go beyond solving local management problems in PENs. The generalization of the intervention was ensured in three different ways: (1) at the level of the structuring of the intervention, (2) at the level of the intervention itself and the analyses carried out, and (3) at the conceptual level.

In terms of structuring the intervention, formative intervention combined local spaces for work and analysis (within each of the PENs), as well as forms of collective grouping at the regional level. In addition, a steering committee for the intervention involved researchers and members of the academic administration, which was set up to draw on this work to lead, criticize, and transform the local implementation of the national priority education policy. This form of intervention structuring aimed to respond to the criticism of weak articulation in CL, in the work grounded in the fourth generation of activity theory (Spinuzzi, 2020a). This structuring aimed to enable the regional discussion of the implementation of public policy from below, while at the same time allowing public policy to guide the work of networks that did not participate in the formative intervention.

At the level of the intervention itself, the work organized in the priority education network groupings differed from the work in boundary-crossing laboratories (Kerosuo and Engeström, 2003; Engeström and Kerosuo, 2007). Indeed, each of the networks was in a situation of independence from the others; the problems faced by each of the networks were distinct, although they sometimes presented similarities. However, the formative intervention did not aim to solve local problems but rather enable actors to redesign their activity system by overcoming identified contradictions. The results showed that the contradictions faced by the workers in PENs were linked to a historically inherited hierarchical structuring that crystallized these power issues. In other words, while the problems were local and singular, the contradictions were general, systemic, and common to all schools participating in the research. It was at this level of analysis that we would like to provide access to the participants. In this context, the comment by a headteacher referring to the Langevin-Wallon plan (see section “Manipulation: Harmonization Versus Achieving One’s Own Goal”) clearly showed that the participants reached this level of generalization beyond the local problems identified.

Finally, at the conceptual level, both the expansion of the object and the practices designed by the participants can be thought of as pilot units that can be generalized (Virkkunen and Newham, 2013). Congruent with this idea was the need to work with academic authorities to support the generalization of practices through the construction of instruments to support the efforts of networks that did not participate in the intervention.

### The Importance of Power Issues in the Emergence of a Fourth Generation of AT

Our work with the priority education networks consisted of getting schools that were not usually used to work together institutionally. The only linkage was found in the student evaluations sent out at the end of the school year. At first glance, one might think that since all schools were part of a largely centralized education system, there might be, beyond the vertical division of labor, a form of homogeneity among the different actors. Our results showed that this is far from the case. The heterogeneity of the different partners involved in formative interventions has its origins in a systemic form of historically inherited power. Within these formative interventions, several functions coexist, sometimes with widely different statuses, several professions, and several institutions. The setting up of PENs raised the question of the construction of a collective activity by partners who are institutionally designated but who are not used to working together. If their activities seem to be neighboring activities, in reality, they are not. The aim of this work remained to set up a collective activity, whose purpose was to reduce educational inequalities well beyond the organization of work maintaining the *status quo* with regard to existing practices. This implied temporal, spatial, and political expansion of the object of their current activity.

From this point of view, this work fits well with the perspective for a fourth generation of activity theory. It is a question of building a coalition of heterogeneous actors around a runaway object (Sannino, 2020). Sannino (2020, p.5) used the term heterogeneous because it means “*qualitatively different types of work and because they operate at different hierarchical levels in the society.*” For us, the signification could be completed. Our results showed that heterogeneous also means “because their hierarchical levels express forms of symbolic domination.” Beyond the different hierarchical levels are the stakes of historically constituted powers. Not all actors have the same freedom of speech and not all words are equally valid. This situation was experienced naturally by the actors. Constituting a coalescing heterogeneous work activity around a runaway object means that the first step must go beyond existing power relations, which seems natural for the actors. In the words of the coordinator in Phase 2 of our formative research, it is a question of going beyond a hierarchical society with which we should live.

To conclude, the developmental intervention presented above allowed the construction of the problem of power relations within a networked organization. It now remains to continue the cycle of expansive learning by enabling professionals to design and experiment with a new form of work that integrates criticism of the vertical division of power.

## Data Availability Statement

The raw data supporting the conclusions of this article will be made available by the authors, without undue reservation.

## Ethics Statement

Ethical review and approval was not required for the study on human participants in accordance with the local legislation and institutional requirements. The patients/participants provided their written informed consent to participate in this study.

## Author Contributions

All authors were involved in the design of the research-intervention, the data collection, and the analysis of the results. The contribution of the authors reflects their effective participation in the writing of the text. The majority of the writing of the text was undertaken by YL.

## Conflict of Interest

The authors declare that the research was conducted in the absence of any commercial or financial relationships that could be construed as a potential conflict of interest.

## Footnotes

1 PISA is an internationally standardized evaluation of educational systems in 30 OECD countries. The purpose of PISA is to assess the extent to which 15-year-olds completing compulsory schooling have the knowledge and skills required to play their full role in society (Figazzolo, 2009, p. 3).
